# Effect of Sodium Chloride on Pyrite Bioleaching and Initial Attachment by *Sulfobacillus thermosulfidooxidans*

**DOI:** 10.3389/fmicb.2020.02102

**Published:** 2020-09-11

**Authors:** Dieu Huynh, Javiera Norambuena, Christin Boldt, Stefan R. Kaschabek, Gloria Levicán, Michael Schlömann

**Affiliations:** ^1^Environmental Microbiology, Institute of Biosciences, TU Bergakademie Freiberg, Freiberg, Germany; ^2^Biology Department, Universidad de Santiago de Chile, Santiago, Chile

**Keywords:** *Sulfobacillus thermosulfidooxidans*, pyrite bioleaching, bacterial attachment, chloride tolerance, microbial iron oxidation

## Abstract

Biomining applies microorganisms to extract valuable metals from usually sulfidic ores. However, acidophilic iron (Fe)-oxidizing bacteria tend to be sensitive to chloride ions which may be present in biomining operations. This study investigates the bioleaching of pyrite (FeS_2_), as well as the attachment to FeS_2_ by *Sulfobacillus thermosulfidooxidans* DSM 9293^T^ in the presence of elevated sodium chloride (NaCl) concentrations. The bacteria were still able to oxidize iron in the presence of up to 0.6M NaCl (35 g/L), and the addition of NaCl in concentrations up to 0.2M (~12 g/L) did not inhibit iron oxidation and growth of *S. thermosulfidooxidans* in leaching cultures within the first 7 days. However, after approximately 7 days of incubation, ferrous iron (Fe^2+^) concentrations were gradually increased in leaching assays with NaCl, indicating that iron oxidation activity over time was reduced in those assays. Although the inhibition by 0.1M NaCl (~6 g/L) of bacterial growth and iron oxidation activity was not evident at the beginning of the experiment, over extended leaching duration NaCl was likely to have an inhibitory effect. Thus, after 36 days of the experiment, bioleaching of FeS_2_ with 0.1M NaCl was reduced significantly in comparison to control assays without NaCl. Pyrite dissolution decreased with the increase of NaCl. Nevertheless, pyrite bioleaching by *S. thermosulfidooxidans* was still possible at NaCl concentrations as high as 0.4M (~23 g/L NaCl). Besides, cell attachment in the presence of different concentrations of NaCl was investigated. Cells of *S. thermosulfidooxidans* attached heterogeneously on pyrite surfaces regardless of NaCl concentration. Noticeably, bacteria were able to adhere to pyrite surfaces in the presence of NaCl as high as 0.4M. Although NaCl addition inhibited iron oxidation activity and bioleaching of FeS_2_, the presence of 0.2M seemed to enhance bacterial attachment of *S. thermosulfidooxidans* on pyrite surfaces in comparison to attachment without NaCl.

## Introduction

Biomining applies sulfur‐ and iron (Fe)-oxidizing microorganisms to extract valuable metals usually from low-grade sulfidic ores and concentrates *via* bioleaching or biooxidation (Rawlings, 2002). Gold processing, recovery of copper, or coal desulfurization used this technology widely (Konishi et al., 1993; Bosecker, 1997). Nowadays, biomining is increasingly applied to process ores of copper, nickel, cobalt, zinc, and uranium (Johnson, 2014; Schippers et al., 2014).

Acidophilic iron‐ and/or sulfur-oxidizing microorganisms play a key role in both natural and industrial processes of sulfide leaching. Iron-oxidizing bacteria, for instance, catalyze ferrous iron (Fe^2+^) oxidation, with ferric iron (Fe^3+^) being regenerated up to ~10^6^ times faster than by chemical autoxidation in acidic environments (Lacey and Lawson, 1970). Thereby, they increase metal dissolution rates considerably. Under environmental acidic conditions, there is a wide range of acidophilic microorganisms capable of oxidizing Fe^2+^ and therefore potentially applicable for biomining (Hedrich et al., 2011). However, acidophilic leaching microorganisms are commonly sensitive to chloride ions (Shiers et al., 2005). *Acidithiobacillus ferrooxidans*^T^, for example, the most widely studied bacterium in bioleaching, tolerates only 6 g/L (~100 mM) sodium chloride (NaCl; Dopson et al., 2017). Biooxidation of Fe^2+^ by a *Leptospirillum ferriphilum*-dominated culture was completely inhibited by 12 g/L chloride (Gahan et al., 2010). Several studies have indicated mechanisms of chloride toxicity in acidophilic bacteria, such as osmotic imbalance, cytoplasmic acidification, and oxidative stress induction (Alexander et al., 1987; Suzuki et al., 1999; Slonczewski et al., 2009; Rivera-Araya et al., 2019). The influx of chloride ions and protons into bacterial cells lowers cytoplasmic pH, disrupts pH homeostasis, and consequently causes cell death for *A. ferrooxidans* and *Acidithiobacillus thiooxidans* (Suzuki et al., 1999). Additionally, chloride tolerance levels were also found to be varied between different domains, genera, or species of acidophilic bacteria (Rea et al., 2015) but commonly, chloride tolerance concentrations are far below seawater levels (Zammit et al., 2012). Rivera-Araya et al. (2017) pointed out that *L. ferriphilum*^T^ is more tolerant to NaCl than *A. ferrooxidans*^T^, with minimum inhibitory concentrations of NaCl being 225 mM (~13 g/L) and 150 mM (~9 g/L), respectively.

While in several studies reasons for the inhibitory effect of chloride on bioleaching organisms have been investigated, almost nothing is known about the effect of chloride on attachment of Fe‐ or sulfur-oxidizing microorganisms to sulfidic minerals. Bacterial attachment is an important step for bioleaching due to the formation of a reaction space between metal sulfide surfaces and bacterial cells (Vera et al., 2013; Bellenberg et al., 2014). A number of researchers have investigated biofilm formation of leaching microorganisms (Rodríguez et al., 2003; Africa et al., 2010; Florian et al., 2011; Bellenberg et al., 2014; Li et al., 2018; Zhang et al., 2019). Attachment and biofilm formation are primarily regulated by quorum sensing and the second messenger cyclic diguanylate c-di-GMP with genes involved in early biofilm formation and c-di-GMP metabolism being identified or upregulated in attached cells of acidophilic bacteria such as *A. ferrooxidans* and *A. thiooxidans* (Mamani et al., 2016; Díaz et al., 2018). Extracellular polymeric substances (EPSs) are known to mediate microbial attachment and biofilm formation, and the synthesis of EPS is stimulated by attachment (Vandevivere and Kirchman, 1993). The bacterial self-produced EPS primarily consist of polysaccharides, proteins, lipids, and DNA (Flemming and Wingender, 2010) with the amount and composition of the EPS varying according to the growth substrates (Gehrke et al., 2001). In case of *A. ferrooxidans*, for example, the complexed iron (III) ions are present in the EPS of pyrite (FeS_2_)-grown cells but not in that of elemental sulfur-grown cells (Gehrke et al., 1998). Despite these insights into attachment and biofilm formation, the effect of chloride on them obviously was not an issue. Whereas, one might expect an inhibitory effect of chloride on attachment, a biofilm could also be triggered as cell response to stress conditions and thus improve bacterial adaptation to unfavorable circumstances (Bellenberg et al., 2019; Bremer and Krämer, 2019). In fact, microorganisms growing in biofilms are more resistant to extreme environments than planktonic counterparts (Hall-Stoodley et al., 2004). Lee et al. (2014) observed that NaCl addition resulted in an increased biofilm formation and overexpressed biofilm-related genes in *Staphylococcus aureus*.

Chloride ions may be present in leaching liquors through the dissolution of gangue materials or from using saline or seawater, etc., and the concentration will be increased by the recycling of the raffinate connected to evaporation of water from the heaps (Bobadilla-Fazzini et al., 2017; Cisternas and Gálvez, 2017). Due to the sensitivity of leaching microorganisms to chloride, bioleaching operations are challenged, especially in those areas where rocks contain high chloride concentrations and where freshwater is scarce such as in Northern Chile or Western Australia. On the other hand, advantages of chemical leaching in the presence of high chloride concentrations have been reported. They include increased solubility and leaching rates (Winand, 1991; Dutrizac, 1992). Positive effects of chloride addition on bioleaching of chalcopyrite (CuFeS_2_) have also been recorded. Thus, Bevilaqua et al. (2013) observed an enhancement of copper dissolution from CuFeS_2_ using *A. ferrooxidans* and the addition of 100 mM NaCl (~6 g/L). In the presence of 3 g/L chloride, chalcopyrite leaching by *S. thermosulfidooxidans* Cutipay was clearly improved (Bobadilla-Fazzini et al., 2014). However, chloride concentrations that proved to be beneficial for bioleaching were very low and the effect of chloride could vary between different metal sulfides (Deveci et al., 2008; Huynh et al., 2019). Therefore, to enable bioleaching in presence of relatively high chloride concentrations and to enable the use of seawater in the mining industry, efforts to search for highly chloride-tolerant leaching microorganisms and studies on bioleaching of metal sulfides in presence of chloride have been made over the past decades (Alexander et al., 1987; Deveci et al., 2008; Gahan et al., 2009; Zammit et al., 2012; Huynh et al., 2017; Norris et al., 2020). Of special interest in this context are bacteria which do not only tolerate chloride but also need the presence of NaCl for growth like *Acidihalobacter properus* or the recently proposed *Acidihalobacter aeolianus* and *Acidihalobacter ferrooxydans* (Khaleque et al., 2019).

Pyrite is the most abundant metal sulfide mineral. Though the economic value of FeS_2_ itself is small, FeS_2_ is frequently associated with gold and many valuable metal sulfides, such as CuFeS_2_, sphalerite (ZnS), and galena (PbS; Chandra and Gerson, 2010). Microbiologically accelerated oxidation of FeS_2_ is not only of environmental concern but it also has economic importance. Microbial pyrite oxidation in active or abandoned coal and metal mines is responsible for acid mine drainage, a serious water pollution problem (Baker and Banfield, 2003). In commercial processes, by generating acidic ferric sulfate solution, pyrite oxidation enhances copper and uranium recovery from ores and wastes (Bruynesteyn, 1989; Bosecker, 1997). Microbial leaching of FeS_2_ has been shown to have potential applications for coal cleaning (Olson and Brinckman, 1986) and has been applied as an important pretreatment step for gold extraction (Kaksonen et al., 2014c). Bioleaching of FeS_2_ with a mixed culture dominated by *L. ferriphilum*, *Acidithiobacillus caldus*, *Acidimicrobium* sp. and *Sulfobacillus* sp., was inhibited by NaCl concentration as low as 4 g/L (Gahan et al., 2009). The presence of 7 or 20 g/L NaCl resulted in an extended lag phase preceding pyrite dissolution (Zammit et al., 2012). Moreover, in the same study, the authors showed that only *S. thermosulfidooxidans*, an *Acidihalobacter* (*Thiobacillus*) prosperus-like strain, and an *Acidiphilum*-like strain were detected from the inoculum after 14 days of bioleaching in the presence of 7 or 20 g/L NaCl (Zammit et al., 2012). Noticeably, Khaleque et al. (2018) indicated the possibility of pyrite bioleaching at a chloride concentration of 30 g/L NaCl using halotolerant acidophilic microorganisms such as *A. aeolianus* (prosperus) DSM 14174 or *A. ferrooxydans* DSM 14175.

Compared to many other acidophilic metal-oxidizing bacteria, commonly found in acidic, sulfur-rich environments and bioleaching operations, *S. thermosulfidooxidans* can tolerate relatively high chloride concentrations (Zammit et al., 2012). Furthermore, *S. thermosulfidooxidans* can oxidize not only Fe^2+^ but also inorganic sulfur compounds (ISC) (Karavaiko et al., 2005). Having the ability to oxidize Fe^2+^ and ISC makes *S. thermosulfidooxidans* a favorable bacterium for heap bioleaching as oxidation of ISC helps to remove excess sulfur compounds and generate the necessary acidity (Dopson and Lindstrom, 1999; Dopson et al., 2009). An earlier study indicated that a chloride concentration of 200 mM inhibited bioleaching of sphalerite by *S. thermosulfidooxidans* but not of CuFeS_2_ (Huynh et al., 2019). Additionally, albeit several studies have been performed on cell attachment of *S. thermosulfidooxidans* to sulfides (Sampson et al., 2000; Ghauri et al., 2007; Li et al., 2016), no investigation on the attachment of *S. thermosulfidooxidans* in the presence of chloride is available. Therefore, this study aims to extend our previous work (Huynh et al., 2019) and investigates if chloride inhibits pyrite bioleaching by *S. thermosulfidooxidans*. In addition to pyrite bioleaching, this study examines the attachment of *S. thermosulfobacillus* cells on pyrite surfaces in the presence of chloride.

## Materials and Methods

### Bacteria and Growth Conditions

*S. thermosulfidooxidans* DSM 9293^T^, a Gram-positive Fe/sulfur-oxidizing bacterium, was used in this study. Bacterial cells were grown in Mackintosh basal salt solution (Mac medium) pH 1.8 (Mackintosh, 1978) with 0.02% yeast extract and 50 mM Fe^2+^ (supplied as FeSO_4_·7H_2_O). Mac medium pH 1.8 and yeast extract were autoclaved separately at 120°C for 20 min, while the ferrous sulfate (FeSO_4_) was sterilized by filtration (0.22 μm, Millipore). Cultures were incubated at 45°C under constant shaking at 120 rpm.

### Pyrite Preparation

Pyrite concentrate with a grain size of 50–100 μm was used (Baia Mare, Romania). The composition of the pyrite concentrate was determined using X-ray powder diffraction analysis, and elemental concentrations were measured by ICP-MS with rhodium and rhenium as internal standards (Wiche and Heilmeier, 2016) after microwave digestion of the pyrite concentrate with nitric acid and hydrochloric acid. The pyrite concentrate contained 84.3% pyrite, 8.1% quartz, 7.6% szomolnokite (FeSO_4_·H_2_O), and ~50 mg Fe/100 mg pyrite concentrate.

The preparation of FeS_2_ was done, as described by Schippers et al. (1999). Briefly, pyrite grains were boiled in 6M HCl for 30 min and then washed with deionized water until the pH was back to neutral. Afterward, grains were stirred in acetone for 30 min in order to remove soluble sulfur compounds. Pyrite samples were kept in a fume hood at room temperature for 12–24 h to evaporate acetone residues. The washed pyrite was stored under nitrogen atmosphere and sterilized dry for 24 h at 120°C.

### Pyrite Bioleaching in the Presence of Sodium Chloride

Bioleaching experiments were carried out in 250-ml baffled Erlenmeyer flasks. Each flask contained 100 ml Mac medium pH 1.8 with a defined NaCl concentration, 0.02% yeast extract, 30 mM Fe^2+^, 5% (w/v) of the washed pyrite concentrate, and 10% (v/v) inoculum of *S. thermosulfidooxidans* pre-culture. NaCl concentrations were 0, 0.1, 0.2, 0.3, 0.4, 0.5, 0.6, and 1M. Incubations were performed at 45°C in a shaking incubator at 120 rpm.

Sampling was done periodically (at days 0, 1, 2, 3, 7, 22, 23, and 36) for monitoring of pH (WTW Sentix 21) and redox potential (vs. Ag/AgCl with 3M KCl; WTW Sentix oxidation-reduction potential, ORP). Leachate samples were also taken for enumeration of planktonic bacteria using a Thoma bacteria counting chamber at 200x magnification. Iron oxidation and pyrite dissolution were followed by determination of the Fe^2+^ iron and total iron concentrations over time by using the Ferrozine method (Braunschweig et al., 2012). The rate of bioleaching was determined from the slope of a curve plotting soluble total iron vs. time using the linear regression function “lm ()” in R (R version 3.6.3) and was reported as mg/L dissolved Fe per hour. Besides, at the end of the experiment (day 36), the bioleached residues were dissolved in 6M HCl for complete dissolution of secondary Fe precipitates while the FeS_2_ remained undissolved. The dissolved precipitates in the residues were then measured for iron concentrations and the iron concentrations in the assays with different amounts of added NaCl were compared. The experiment was done in triplicate and data were presented as means ± SD.

In addition, chemical leaching of FeS_2_ without bacteria by Fe^3+^ in the presence of NaCl was also performed. The experiment was carried out in 250-ml baffled Erlenmeyer flasks containing 100 ml Mac medium pH 1.8 with a defined NaCl concentration (0, 0.1, 0.2, 0.3, 0.4, 0.5, 0.6, or 1M). Iron addition was in the concentration of either ~40 mM Fe^3+^ (supplied as Fe_2_(SO_4_)_3_·xH_2_O) or 20 mM Fe^3+^ and 30 mM Fe^2+^ (supplied as FeSO_4_·7H_2_O). Incubations were at 45°C in a shaking incubator at 120 rpm. Samples were taken periodically for determination of iron, pH, and redox potential. The experiments were done in duplicate.

### Bacterial Attachment in the Presence of NaCl

Experiments on bacterial attachment to pyrite grains were carried out in duplicate and performed in 100-ml Erlenmeyer flasks. Each flask was filled with 20 ml Mac medium pH 1.8 containing a specified NaCl concentration and 1 g pyrite grains (50–100 μm), and it was inoculated with *S. thermosulfidooxidans* to an initial concentration of 10^8^ cells/ml. Cells of *S. thermosulfidooxidans* grown on iron in absence of NaCl (Mac medium pH 1.8, 0.02% yeast extract, and 50 mM Fe^2+^) were harvested by centrifugation at 10,000 rpm for 15 min and washed twice with Mac medium pH 1.8 before inoculation. NaCl concentrations were 0, 0.2, and 0.4M. No exogenous iron was added into the inoculated flasks to avoid the occurrence of iron precipitation due to biooxidation of Fe^2+^. The cultures were shaken at 120 rpm in a rotary shaker at 45°C. Samples of FeS_2_ were taken after 18 h of incubation for attachment visualization and quantification of sessile cells.

#### Microscopy Sample Preparation

Pyrite grains were first gently rinsed three times with sterile-filtered water and cells on pyrite surfaces were fixed with 2.5% glutaraldehyde for 1 h. Afterward, pyrite grains were rinsed three times with sterile water and stained in 6 μM SYTO 9 (Invitrogen) for 15 min. Stained pyrite grains were then gently washed another three times with sterile-filtered water to remove unbound dye before visualization. Direct light exposure was avoided (modified after Li et al., 2016; Schieferbein et al., 2017).

#### Visualization and Image Analysis

The cell attachment on pyrite surfaces was visualized by confocal laser-scanning microscopy (CLSM) using an upright Zeiss LSM 800. Stained mineral grains were mounted on diagnostic glass slides (8-well, 6.7 mm; Thermo Scientific) without cover lip and were recorded as stack images using the non-immersion objective EC Epiplan-Apochromat 50x/0.95 HD DIC M27. Laser excitation wavelength was 488 nm, and emission wavelengths were at 525 nm, respectively. The pyrite surface was visualized by CLSM in reflection mode. The ZEN Blue 2.6 (Carl Zeiss GmbH) imaging software was used for data acquisition and the images were displayed in *ortho* view with maximum intensity projection and exported as JPEG format. Areas of attached cells and pyrite grains were determined from 7–8 images per each condition using ImageJ. Bacterial attachment on pyrite surfaces was estimated based on the percentage of cell area vs. pyrite surface area. Statistical analysis was carried out by a Kruskal-Wallis test, a non-parametric alternative to the one-way ANOVA test, and pairwise comparisons between different NaCl concentrations were done using the Wilcoxon test.

### Quantification of Sessile Cells by Quantitative PCR

#### DNA Preparation

Samples of FeS_2_ from the bacterial attachment experiment in the presence of NaCl (as described above) were also used for DNA extraction. Briefly, liquids were carefully removed from flasks and pyrite samples were air-dried for at least 1 h. Exactly 0.8 g of pyrite grains was weighed to a 1.5-ml Eppendorf tube and 50 μl of 10 mg/ml lysozyme solution was added into the pyrite-containing tube. The content of the tubes was gently mixed and then incubated for 30 min at 37°C for cell lysis. Afterward, DNA extraction was performed using a DNeasy UltraClean Microbial Kit (Qiagen) as per manufacturer’s description.

#### PCR Primers and qPCR Analysis

A specific section of the *gyrase B* gene from *S. thermosulfidooxidans*, of which it contains one copy, was amplified using the primers sulfo_gyrB_fwd (5'-CGGCGATTGTTTCGGTTAAAT-3') and sulfo_gyrB_rev (5'-CCATCGGCGGTAATTCCTTC-3') and cloned into pSC-A-amp/kan (StrataClone PCR cloning kit; Agilent). Plasmids were purified with the GeneJET Plasmid Miniprep Kit (Thermo Scientific) and quantified using a NanoDrop_ND-1000 spectrophotometer (NanoDrop Technologies). A calculator from the URI Genomics & Sequencing Center^1^ was used to determine the copy number of the plasmid. Then, the prepared plasmid was diluted serially from 10^8^ to 10^3^ copies/μl and quantitative PCR (qPCR) was performed in the Rotor-Gene 3000 (Corbett Research) to generate a calibration curve of Ct (threshold cycle) vs. the number of gene copies. The extracted DNA from attached cells of *S. thermosulfidooxidans* was amplified by qPCR using a reaction mixture containing 5 μl PowerUp SYBR Green Master Mix (Applied Biosystems), 0.625 μl of 10 μM primer sulfo_gyrB_for and 1.25 μl of 10 μM primer sulfo_gyrB_rev, 1 μl template DNA, and water added to a total volume of 10 μl. The qPCR program was performed as follows: 55°C for 1 min, 95°C for 5 min, and 40 cycles of 95°C for 10 s, 60°C for 20 s, and 72°C for 20 s. Each DNA extract was measured in triplicate. Different extents of cell adhesion on mineral surfaces at various NaCl concentrations were indirectly compared based on the number of gene copies/(μl template DNA) found under each condition in the DNA isolated from the attached cells.

## Results

### Bioleaching of Pyrite in the Presence of Sodium Chloride

**Figure 1:**
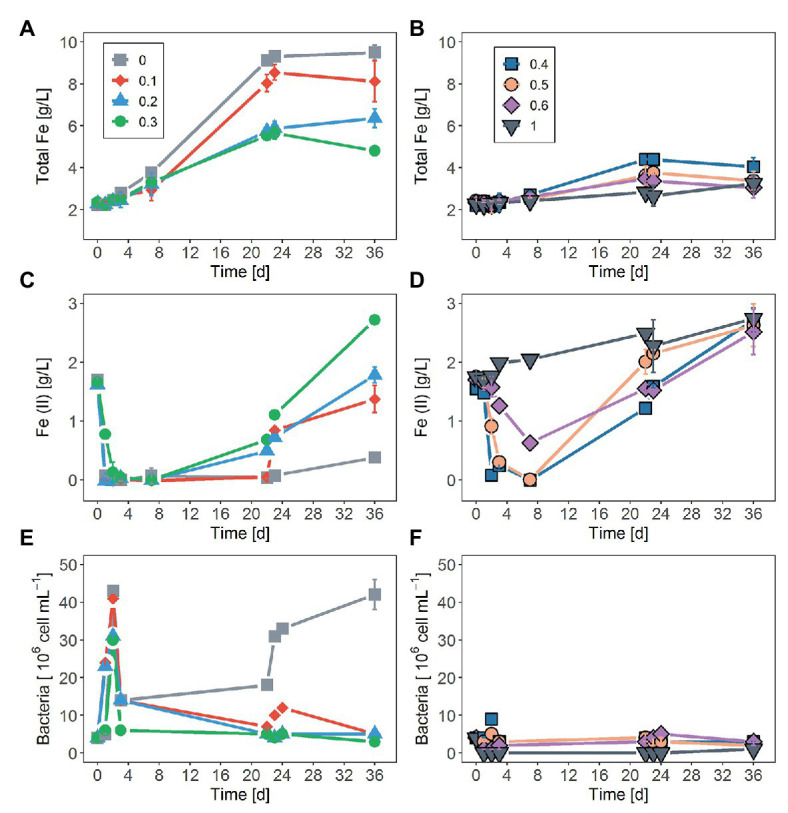
Effect of sodium chloride (NaCl) on: **(A,B)** total iron concentration, **(C,D)** changes in the ferrous ion (Fe^2+^) concentration, and **(E,F)** planktonic cell numbers as a function of time during the bioleaching of pyrite (FeS_2_) using *Sulfobacillus thermosulfidooxidans*. Concentrations of NaCl were 0.1, 0.2, 0.3, 0.4, 0.5, 0.6, and 1M. The control assay contained medium without addition of NaCl. The data represent the mean of three replicates ± standard deviation.

**Figure 2:**
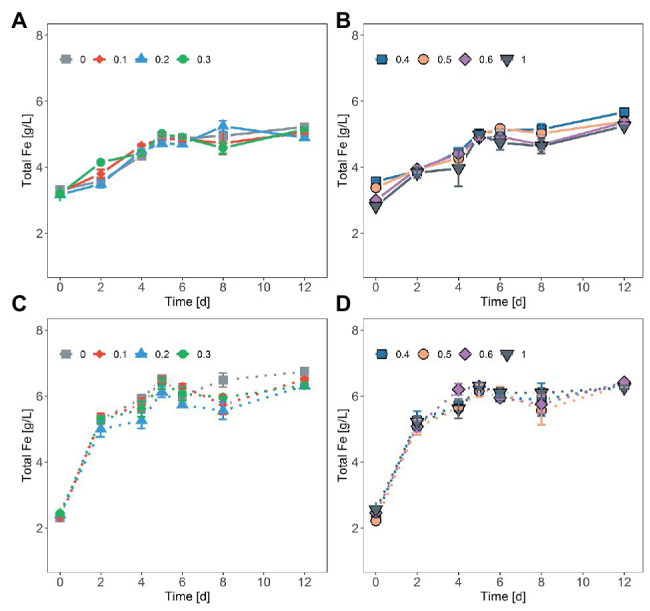
Chemical oxidation of FeS_2_ in the presence of NaCl by ferric iron (Fe^3+^) concentrations of ~20 mM Fe^3+^ and 30 mM Fe^2+^ (solid line, **A,B**) or ~40 mM Fe^3+^ (dash line, **C,D**). NaCl concentrations were 0.1, 0.2, 0.3, 0.4, 0.5, 0.6, and 1M. The data represent the mean of duplicate experiments ± deviations from the means.

In tests on pyrite bioleaching with cells of *S. thermosulfidooxidans* at various concentrations of NaCl, it was shown that the addition of NaCl reduced pyrite dissolution. The highest bioleaching performance was obtained without NaCl, with nearly 7 g/l Fe (~28%) being leached after 23 days (Figures 1A,B). The inhibitory effect of NaCl on bioleaching of FeS_2_ was observed already at a NaCl concentration as low as 0.1M when only around 85% of the total dissolved Fe of the leaching without NaCl was reached. Bioleaching with 0.2 and 0.3M NaCl gave 66 and 50%, respectively, of the total Fe dissolved in the absence of NaCl. The yield of pyrite bioleaching with 0.5M was as low as the bioleaching in the presence of 0.6M NaCl. Pyrite leaching was completely inhibited by the addition of 1M NaCl. After 23 days of leaching, regardless of the NaCl concentration, almost no further leaching activity occurred. Not only the degree of pyrite leaching but also the pyrite dissolution rate was significantly reduced in the presence of NaCl. The higher NaCl concentrations showed lower pyrite dissolution rates. Under the conditions investigated, the highest pyrite dissolution rate (13 mg Fe/L/h) was obtained in the absence of NaCl, followed by the 0.1M NaCl (Figures 1A,B and Table 1). In chemical leaching assays, a plateau was reached earlier and at a lower iron concentration. NaCl concentrations showed almost no effect on the rate of pyrite dissolution while lower concentration of iron (III) obviously resulted in the lower dissolution of FeS_2_ (Figure 2).

**Table 1:** Dissolution rate (mgL^−1^ h^−1^) for pyrite bioleaching by *S. thermosulfidooxidans* with varying concentrations of sodium chloride.

	NaCl concentration (M)							
	0	0.1	0.2	0.3	0.4	0.5	0.6	1
Fe dissolution rate (mgL^−1^ h^−1^)	13.46	11.17	7.03	5.81	3.89	2.47	1.96	nd
Fit (R^2^)	1.00	0.97	0.92	0.97	0.96	0.86	0.88	nd

The rate was determined over the linear leaching period. The table summarizes data shown in Figures 1A,B. Fit (R^2^), the coefficient of determination indicates how well the linear regressions fit the experimental data. nd, no leaching and the Fit R^2^ < 0.8.

As shown in Figures 1C,D, within the first 7 days of the experiment, Fe^2+^ oxidation was not significantly inhibited by NaCl concentrations lower than 0.3M while a clear decline in biological oxidation was recorded in the presence of NaCl concentrations higher than 0.3M. The initially added external Fe^2+^ was completely oxidized within 1 day in assays without NaCl, with 0.1 and 0.2M NaCl, whereas in assays with 0.3M NaCl complete Fe^2+^ oxidation required ca. one additional day. More than 3 days were necessary for complete Fe^2+^ oxidation in the presence of 0.4 and 0.5M NaCl. At 0.6M NaCl after a lag phase of roughly 2 days, only partial Fe^2+^ oxidation occurred. Iron oxidation activity of *S. thermosulfidooxidans* was completely suppressed at 1M NaCl.

Regarding changes in numbers of planktonic cells during bioleaching duration, an increase in NaCl concentration above 0.1M led to a decrease in planktonic cell counts (Figures 1E,F). After an initial rise within the first 2 days, planktonic cell counts dropped drastically and remained relatively low in assays with addition of 0.1–0.3M NaCl over duration of the experiment. A similar pattern was observed without NaCl. However, in this case, planktonic cells continued to develop after 22 days. At NaCl concentrations of 0.4 and 0.5M, very little growth was observed at day 2, followed by a loss in planktonic cells by day 4 and low concentrations until the end of the experiment. No planktonic cell growth was detected in the presence of 0.6 and 1M NaCl.

Since in the presence of 0.1–0.2M NaCl, *S. thermosulfidooxidans* was still able to oxidize Fe^2+^ to Fe^3+^ with a considerable rate, it reached high ORP values. In the presence of 0, 0.1, and 0.2M NaCl, ORP increased from approximately 450 mV to ca. 610 mV within 3 days (Supplementary Figure S1). The addition of 0.3M caused a slight decrease in ORP development compared to ORP in assays with NaCl concentration <0.3M. After addition of NaCl concentrations >0.3M, the ORP in the first few days rose even less. After day 3, in the absence of NaCl, the ORP remained high at approximately 620 mV until day 22 and then dropped to around 540 mV. A decline of ORP after the initial increase was also observed in the presence of NaCl. The pH value in the absence and presence of up to 0.5M NaCl increased at beginning of the experiment and started to decrease after 2 days of leaching experiment. Above 0.5M NaCl, there was no increase but just some decrease of the pH (Supplementary Figure S1). For chemical leaching of FeS_2_ by Fe^3+^, at all investigated concentrations of NaCl ORP and pH experienced a significant decrease at day 2, followed by a gradual decrease until Fe^3+^ was completely reduced to Fe^2+^ (Supplementary Figure S2).

**Figure 3:**
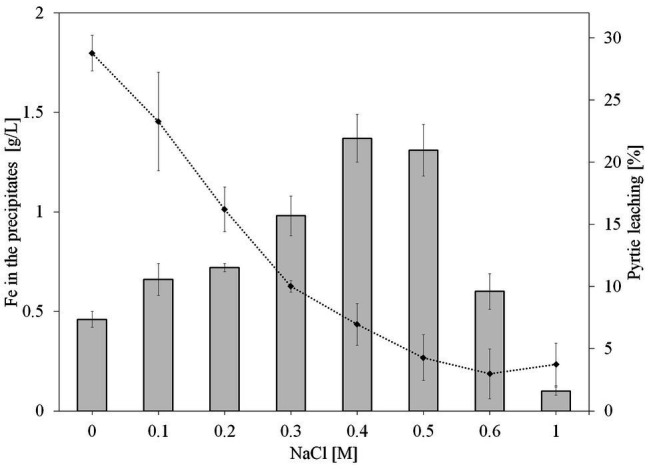
Effect of NaCl on iron concentration in the leaching precipitates (bar graph) and leaching efficiency (line graph) after 36 days of pyrite bioleaching by *S. thermosulfidooxidans*. NaCl concentrations were 0, 0.1, 0.2, 0.3, 0.4, 0.5, 0.6, and 1M. The error bars represent the standard deviation of the three replicates.

At the end of the experiment (day 36), the leaching solution was removed and the precipitate was dissolved with 6M HCl and the iron concentration was measured. Considering the absolute amounts of precipitated iron divided by the culture volumes, in the absence of NaCl, approximately 0.5 g/L Fe had been precipitated, while the addition of 0.1–0.6M NaCl had resulted in even higher iron precipitation. As shown in Figure 3, the assays with addition of 0.4 and 0.5M NaCl had the highest iron concentration in the precipitate (~1.5 g/L), followed by 0.3M NaCl with ~1 g/L Fe, while cultures with 1M NaCl had the lowest (~<0.1 g/L).

### Bacterial Attachment on Pyrite Surfaces in the Presence of NaCl

**Figure 4:**
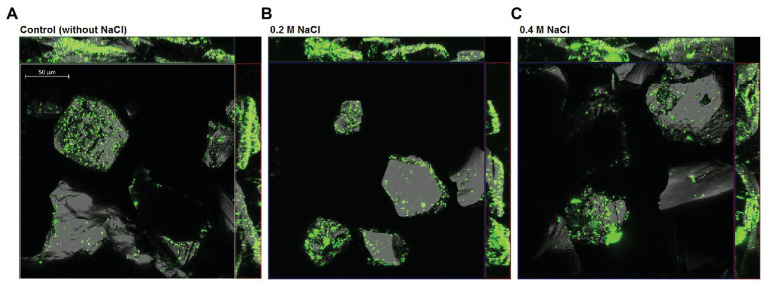
Confocal laser scanning microscopy (CLSM) of pyrite-attached cells of *S. thermosulfidooxidans* after 18 h exposure to 0M **(A)**, 0.2M **(B)**, and 0.4M NaCl **(C)**. Attached cells were stained directly on the pyrite grains using the nucleic-acid dye Syto 9. The size bar measures 50 μm.

As shown in Figure 4, cells of *S. thermosulfidooxidans* attached unevenly on pyrite surfaces, some of the areas on a grain or some grains were very well colonized, whereas other areas or grains remained free of attached bacteria. Moreover, it was observed that more cells attached at surface defects than at smooth surfaces. A good colonization of cells on FeS_2_ was detected in both the absence of NaCl and the presence of 0.2M NaCl. Pyrite grains incubated with cells of *S. thermosulfidooxidans* and 0.4M NaCl also showed a presence of attached cells.

**Figure 5:**
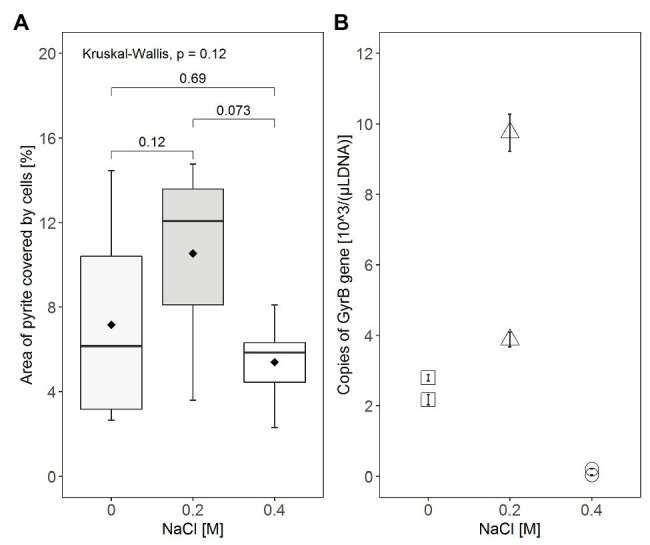
Comparison of attachment on pyrite grains by *S. thermosulfidooxidans* at NaCl concentrations of 0, 0.2, and 0.4M. **(A)** The area of FeS_2_ covered by cells was quantified directly from CLSM images using ImageJ, with the box, the horizontal line, and the point (◆) inside the box representing the middle 50%, the median, and the mean of the distribution of the area of coverage, respectively. The minimum (lower end of vertical line), the maximum (upper end of vertical line), and the 25% (lower end of the box) and 75% (upper end of the box) percentiles of area of coverage are also indicated in the boxplot diagram. Statistical analysis was carried out by Kruskal-Wallis and Wilcoxon tests. **(B)** As an indicator for the number of attached cells, the *gyrB* gene was amplified by quantitative PCR (qPCR) with DNA extracted from cells attached to the pyrite grains. Attachment tests were done in duplicate and qPCR was done in triplicate for each DNA template. Values displayed are the average of triplicated qPCR results for each DNA sample ± standard error.

In addition to illustration of attachment by visualization, attempts were made to quantify attachment of cells to FeS_2_ by image analysis using ImageJ and by qPCR targeting the *gyrB* gene of attached *S. thermosulfidooxidans* cells (Figure 5). As shown in the boxplots (Figure 5A), in the absence of NaCl for most of the pyrite minerals, the bacteria covered areas between 3 and 10%, but on some grains just 2.5 and or even 14%. The boxplot for attachment with 0.2M NaCl shows that under this condition, the bacteria covered between 3.5 and 15% of the pyrite surface with half of the pyrite minerals having a bacterial area coverage between 8 and 14%. By contrast, a lower bacterial attachment was observed in the presence of 0.4M NaCl with most of pyrite surfaces being covered by only 5–6%. Thus, while the variation between replicates was big, the addition of NaCl, especially 0.2M NaCl seems to have resulted in differences in attachment of *S. thermosulfidooxidans* with median area coverage being ~6.1, 12, and 5.8% in the absence of NaCl, the presence of 0.2M, and the presence of 0.4M NaCl, respectively.

Additionally, the effect of NaCl on pyrite attachment of *S. thermosulfidooxidans* was indirectly quantified by qPCR based on the copy numbers of the *gyrB* gene obtained from DNA of attached cells (Figure 5B). Although there was a large variation between the replicate assays among the three conditions compared, assays with the presence of 0.2M NaCl seemed to have the highest attachment of cells with 3.8·10^3^ or 9.7·10^3^ *gyrB* gene copies/(μl template DNA; Figure 5B). In contrast, we found only 2.1·10^3^ or 2.7·10^3^ *gyrB* gene copies/(μl template DNA) in samples without exposure to NaCl and less than 1·10^2^ *gyrB* gene copies/(μl template DNA) were present in samples exposed to 0.4M NaCl.

## Discussion

### Bioleaching of Pyrite in the Presence of Sodium Chloride

*S. thermosulfidooxidans* is more tolerant to NaCl than many other acidophilic iron-oxidizing bacteria used in the mining industry (Zammit et al., 2012; Rea et al., 2015), which make it potentially useful for bioleaching in saline environments. The presence of sodium chloride inhibited bioleaching of sphalerite by *S. thermosulfidooxidans* while bioleaching of CuFeS_2_ was not affected (Huynh et al., 2019). To extend our previous work (Huynh et al., 2019), bioleaching of FeS_2_ by *S. thermosulfidooxidans* with elevated NaCl concentrations was investigated. Although bacteria used for the pyrite bioleaching in this present study differed from the study of Gahan et al. (2010), our study also found that the presence of chloride in pyrite leaching solutions severely affects the leaching yields. The finding indicates that chloride presence inhibits pyrite bioleaching by *S. thermosulfidooxidans*. Nevertheless, the concentrations of NaCl that inhibited bioleaching of FeS_2_ by *S. thermosulfidooxidans* were much higher than the concentrations that inhibited pyrite bioleaching by a *L. ferriphilum*-dominated culture, as reported by Gahan et al. (2010). *S. thermosulfidooxidans* was still able to bioleach pyrite at 0.4M NaCl (~23 g/L) which is already 80% of the concentration of chloride in seawater (~19.4g/L or 0.55M).

Gahan et al. (2009) proposed that the lower pyrite leaching yields must be attributed to the toxicity of chloride on microorganisms and to the precipitation of jarosite. The increase of pH together with the generation of Fe^3+^ during the first 3 days of the experiment could have promoted the precipitation of Fe^3+^ ion. As seen, Fe^3+^ precipitation was enhanced with increasing NaCl concentration up to 0.4M NaCl (Figure 3). This could also be the reason for the decrease in pyrite bioleaching by *S. thermosulfidooxidans* at NaCl concentrations higher than 0.3M, especially since jarosite formation requires the presence of monovalent cations, which would be supplied by the sodium ions. Also, the co-precipitation with important ions for bacterial growth such as phosphate (Nazari et al., 2014) might affect bioleaching during a long operation period.

Iron oxidation of *S. thermosulfidooxidans* was not significantly inhibited within the first 7 days of exposure to ≤0.2M (~12 g/L) NaCl. In contrast, a clear inhibitory effect of chloride on *S. thermosulfidooxidans* was observed at NaCl concentrations of 0.3–1M (17–58 g/L). A previous study (Huynh et al., 2019) had shown that *S. thermosulfidooxidans* could not oxidize Fe^2+^ completely in the presence of 0.5M NaCl (29 g/L) and 0.6M NaCl (35 g/L). However, the present study found an improvement of Fe^2+^ oxidation upon exposure to a similar NaCl concentration. Cells exposed to 0.5M NaCl could oxidize Fe^2+^ fully over the first 2–3 days of the leaching study and Fe^2+^ oxidation still occurred in the presence of 0.6M NaCl, albeit no complete oxidation was observed. Also, though numbers of planktonic cells decreased due to inhibition of growth and/or precipitation, the cells remained active with respect to iron oxidation for several days. This suggests that the presence of FeS_2_ could possibly have improved Fe^2+^ oxidation of *S. thermosulfidooxidans* at an early stage of the oxidation period. Nevertheless, iron oxidation activity of *S. thermosulfidooxidans* was reduced with increasing incubation time characterized by an increase in Fe^2+^ concentration between days 7 and 36 of the leaching experiment. It is currently widely accepted that bacteria oxidize FeS_2_ *via* the thiosulfate mechanism (Sand et al., 1995, 2001; Schippers and Sand, 1999). Bacteria accelerate pyrite oxidation by regenerating Fe^3+^ which oxidizes FeS_2_ in an abiotic reaction and consequently is reduced back to Fe^2+^. The accumulation of Fe^2+^ in leaching solutions, hence, is evidence for insufficient or absent bacterial iron oxidation activity. This trend was especially obvious in the bioleaching assays with elevated NaCl concentrations and up to 0.6M NaCl it looks like the higher the NaCl concentration, the more Fe^2+^ concentration was obtained in the bioleaching solution at the end of the experiment.

High ferric iron/ferrous iron ratios or high redox potentials favor pyrite oxidation and at or below 650 mV (vs. standard hydrogen electrode, ~430 mV Ag/AgCl) bacteria have limited impact on pyrite dissolution (Liu et al., 2017; Borilova et al., 2018). High chloride concentrations inhibit iron oxidation activities of *S. thermosulfidooxidans*, consequently reduce redox potential and therefore prohibit pyrite dissolution. The Fe^2+^ oxidation activity was reduced over the time course of the experiment with and without NaCl being present. Iron oxidation activity in the presence of NaCl, however, was inhibited earlier than in the absence of NaCl. *S. thermosulfidooxidans* are not able to fix CO_2_ efficiently when grow with air (Clark and Norris, 1996; Tsaplina et al., 2000) and oxidize iron poorly under autotrophic conditions (Tsaplina et al., 2000). Supplied yeast extract (0.02%) is considered to act as carbon source for iron-oxidizing heterotrophic bacteria (Bacelar-Nicolau and Johnson, 1999) and it may also be a source of vitamins, like vitamin B12, which can act against oxidative stress to which iron oxidizers are exposed especially in presence of NaCl (Ferrer et al., 2016; Rivera-Araya et al., 2019). Yeast extract can be exhausted over time and this may lower iron oxidation activities of *S. thermosulfidooxidans*. It can be speculated that the concurrence of the carbon source exhaustion and NaCl stress results in a more severe inhibitory effect on growth and iron oxidation even at a concentration as low as 0.1M, and therefore could lessen pyrite bioleaching by *S. thermosulfidooxidans* after 20 days of leaching duration.

In addition, during pyrite oxidation, a wide range of sulfur compounds can be formed as intermediates such as thiosulfate (S_2_O_3_^2−^) and tetrathionate (S_4_O_6_^2−^; Schippers et al., 1996; Schippers and Sand, 1999; Vera et al., 2013). These reduced forms of sulfur are substrates for *S. thermosulfidooxidans* as it can oxidize not only iron but also sulfur compounds (Karavaiko et al., 2005). Several studies have reported that iron oxidation is more sensitive than sulfur oxidation to inhibitory effects by chloride and salt (Harahuc et al., 2000; Bonnefoy et al., 2018). Therefore, exposure to chloride possibly affects the preference for substrate utilization of *S. thermosulfidooxidans*, so that sulfur compounds may be preferred over Fe^2+^. This may lead to additional accumulation of Fe^2+^ and oxidation of the reduced sulfur compounds may increment the extent of acidity. The formation of jarosites also additionally acidifies leaching solutions (Kaksonen et al., 2014a,b). As seen, after an initial increase, pH values gradually dropped to approximately 1.4 in assays with up to 0.5M NaCl (Supplementary Figure S1). The decrease of pH values enhances the toxic effect of chloride on *S. thermosulfidooxidans* (data unpublished; Falagán and Johnson, 2018). Under such pyrite leaching conditions, the inhibition for bacterial growth possibly would become greater and as a result Fe^2+^ oxidation activity of *S. thermosulfidooxidans* could also be increasingly inhibited.

Overall, iron oxidation activity of *S. thermosulfidooxidans* seems to be lower over a long duration of pyrite leaching and the extent of the reduction depends on NaCl concentrations. Pyrite bioleaching is strongly correlated to iron oxidation activity while during the pyrite-dissolution process iron is no longer the only substrate available for *S. thermosulfidooxidans* and carbon sources can also become limiting. Therefore, to improve pyrite bioleaching with NaCl by *S. thermosulfidooxidans*, it is essential to have further investigations on the effect of NaCl on substrate utilization of *S. thermosulfidooxidans* during pyrite bioleaching.

### Effect of NaCl on Pyrite Colonization by *S. thermosulfidooxidans*

Attachment and biofilm formation are important not only for bioleaching of metal sulfides but also for microorganisms to survive environmental stresses. NaCl negatively affects cell growth and Fe^2+^ oxidation activity of leaching microorganisms, including *S. thermosulfidooxidans*. However, details on the bacterial attachment in the presence of NaCl have not been investigated yet. Our study focuses on attachment of cells on pyrite grains at 18 h incubation. The 18 h incubation was chosen for the attachment study due to the low attachment ability of *S. thermosulfidooxidans* to FeS_2_ on the one hand, as well as to the occurrence of cell detachment over long periods (e.g., after 48 h) of incubation on the other (Becker et al., 2011; Li et al., 2016). Unlike *A. ferrooxidans*, where maximum attachment may be obtained within 5–10 min, *S. thermosulfidooxidans* requires days for cell attachment (data not shown; Harneit et al., 2006; Li et al., 2016). Many studies evaluated the attachment of cells by indirect determination of planktonic cell numbers (Porro et al., 1997; Rodríguez et al., 2003; Vardanyan et al., 2019). Exposure to high NaCl concentrations can cause cell death, reduce the number of planktonic cells in suspension and thus it possibly results in overestimation of adhesion cell numbers by such indirect determination. Therefore, by visualization of cell attachment using CLSM, by quantification of attachment area using ImageJ, and by quantitative PCR of attached cells, we attempted to recognize attachment directly as well as to compare the extent of adhesion on pyrite grains of cells exposed to different NaCl concentrations.

Attachment of bacteria to sulfide surfaces is attributed to electrostatic interactions, by which positively charged cells attach to the negatively charged pyrite surface (Blake et al., 1994; Vera et al., 2013). Our observations on attachment of *S. thermosulfidooxidans* on FeS_2_ are in good agreement with Li et al. (2016) and Becker et al. (2011) who reported that the cells of *S. thermosulfidooxidans* were distributed heterogeneously on pyrite surfaces. A similar distribution of cells was observed in the absence of NaCl and with 0.2 or 0.4M NaCl. Cells seemed to adhere preferably at areas around the cracks or defects of the mineral surface (Figure 4). This phenomenon has previously been found to be typical of attachment and biofilm formation of leaching bacteria, colonizing imperfect areas of minerals better than the more perfect areas (Sanhueza et al., 1999; Sand et al., 2001). As reported in a previous study (Huynh et al., 2019), the cell viability of *S. thermosulfidooxidans* was not significantly restricted after exposure to 0.2M NaCl for 18 h. In the present CLSM study, 0.2M NaCl also did not show an inhibitory effect on attachment of *S. thermosulfidooxidans*.

The qPCR results displayed higher copy numbers of *gyrB*/(μl template DNA) for DNA extracted from attached cells with presence of 0.2M NaCl than from cells with the absence of NaCl or 0.4M NaCl. This result appears to be substantiated by a relatively good correlation between the qPCR results and attachment results obtained from CLSM image analysis. Therefore, it can be postulated that NaCl concentration of 0.2M is likely to enhance cell attachment of *S. thermosulfidooxidans* on FeS_2_. The qPCR results indicated that the number of sessile cells after the exposure to 0.4M NaCl was reduced to approximately 94% of the sessile cells in the absence of NaCl. A correlation between pyrite dissolution and initial attachment was not evident in the study. Despite having a similar or to some extent less attachment on FeS_2_ without NaCl than attachment with 0.2M NaCl, pyrite dissolution showed the highest yield in the absence of NaCl. Obviously, the limiting factor in the present case is not the attachment but the iron oxidation. It is likely that the inhibition of microbial Fe^2+^ oxidation by high chloride concentrations, as visible in the Fe^2+^ accumulation, is responsible for lowering pyrite bioleaching.

The occurrence of attachment in the presence of NaCl possibly implies that attachment might play more important roles for salt stress responses than for bioleaching of FeS_2_. In fact, bacterial attachment initiates biofilm formation which could be induced as a stress response. It is generally acknowledged that growth as biofilm can be considered a stress response mechanism: thus, the biofilm mode of growth allows bacteria to increase proliferation, promote survival and propagation of the cells (Jefferson, 2004). Philips et al. (2017) reported NaCl stress-induced biofilm formation of *Clostridium ljungdahlii*, and copper stress-promoted biofilm formation was observed in *S. aureus* (Baker et al., 2010). Proteomic evidence showed that in comparison to planktonic iron-grown cells, pyrite-grown biofilm cells of *A. ferrooxidans* have enhanced oxidative stress responses and metabolic adaptation to oxidative stress (Bellenberg et al., 2019). Moreover, bacteria in biofilms exhibit phenotypical differences from free cells; ATP levels, for instance, were found to be higher in attached cells than in planktonic cells of *A. ferrooxidans* grown on elemental sulfur (Tao and Dongwei, 2014). Therefore, it can be speculated that attachment of *S. thermosulfidooxidans* to FeS_2_ may be part of the stress responses upon exposure to NaCl, a hypothesis which needs to be further investigated.

## Data Availability Statement

The raw data supporting the conclusions of this article will be made available by the authors, without undue reservation.

## Author Contributions

DH designed and performed the experiments and wrote the manuscript. JN and CB contributed to qPCR analysis. GL, MS, and SK contributed to the outline of the study and the redaction of the manuscript. All authors contributed to the article and approved the submitted version.

### Conflict of Interest

The authors declare that the research was conducted in the absence of any commercial or financial relationships that could be construed as a potential conflict of interest.

## Footnotes

^1^https://cels.uri.edu/gsc/cndna.html
