# Supplementary material for: Effect of Sodium Chloride on Pyrite Bioleaching and Initial Attachment by Sulfobacillus thermosulfidooxidans
Source: Front Microbiol. 2020 Sep 11;11:2102. doi: 10.3389/fmicb.2020.02102 (PMC7516052; doi:10.3389/fmicb.2020.02102)
Supplement: Supplementary file 1 [file Data_Sheet_1.docx]

Effect of sodium chloride on pyrite bioleaching and initial attachment by *Sulfobacillus thermosulfidooxidans*

Dieu Huynh^1^*, Javiera Norambuena^1^, Christin Boldt^1^, Stefan R Kaschabek^1^, Gloria Levicán^2^, and Michael Schlömann^1^*

^1^Environmental Microbiology, Institute of Biosciences, TU Bergakademie Freiberg, Freiberg, Germany

^2^Biology Department, Universidad de Santiago de Chile, Santiago, Chile

*** Correspondence:**Ngoc Dieu Huynh
Ngoc-Dieu.Huynh@ioez.tu-freiberg.de

Michael Schlömann

Michael.Schloemann@ioez.tu-freiberg.de


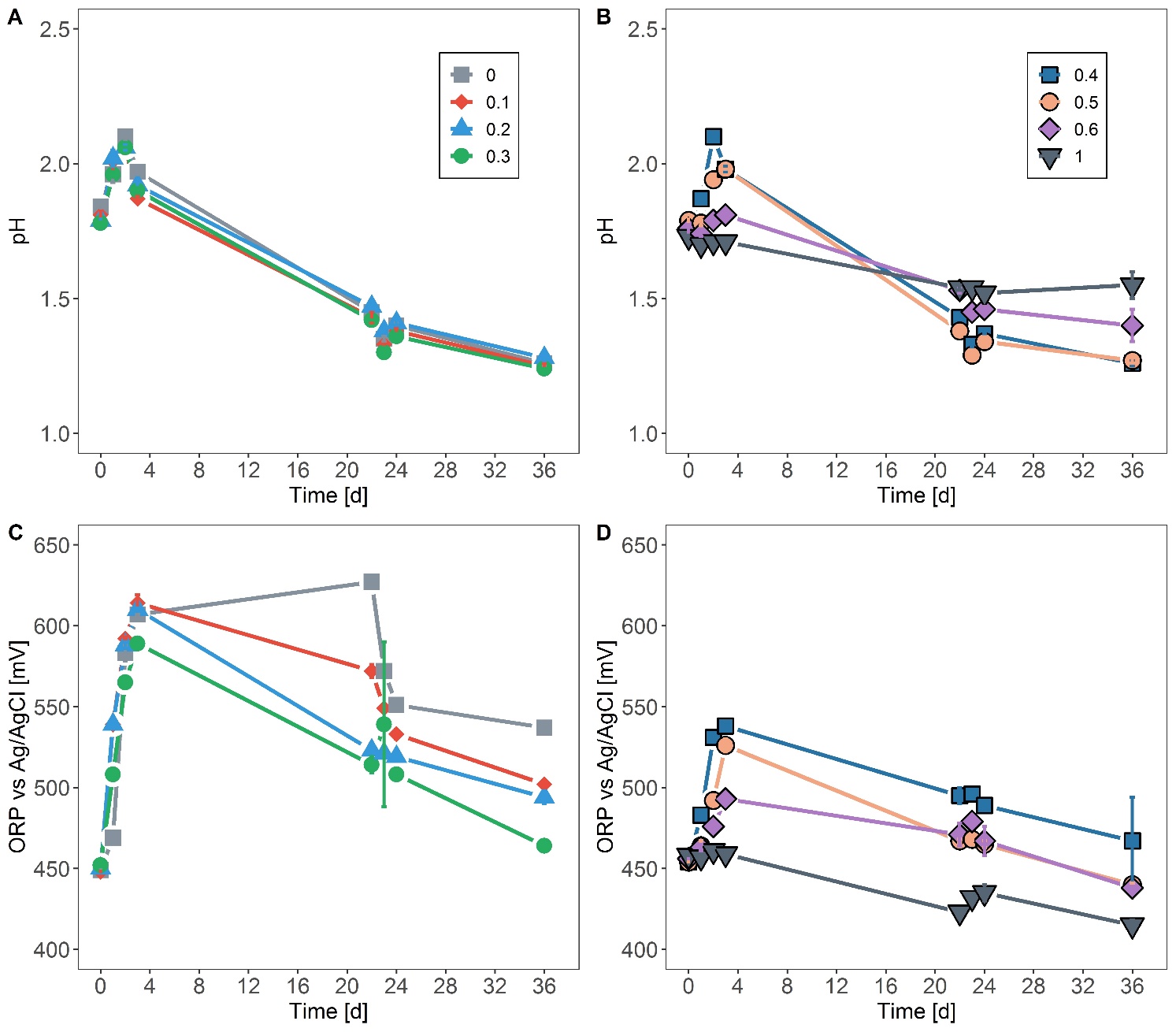


**Supplementary Figure S1**. Changes in pH and ORP (mV vs Ag/AgCl) as a function of time during the bioleaching of pyrite using *S. thermosulfidooxidans* in the presence of different NaCl concentrations. Concentrations of NaCl were 0.1, 0.2, 0.3, 0.4, 0.5, 0.6, and 1M. The control assays contained medium without addition of NaCl. Values are the average of the triplicate ± standard deviation.


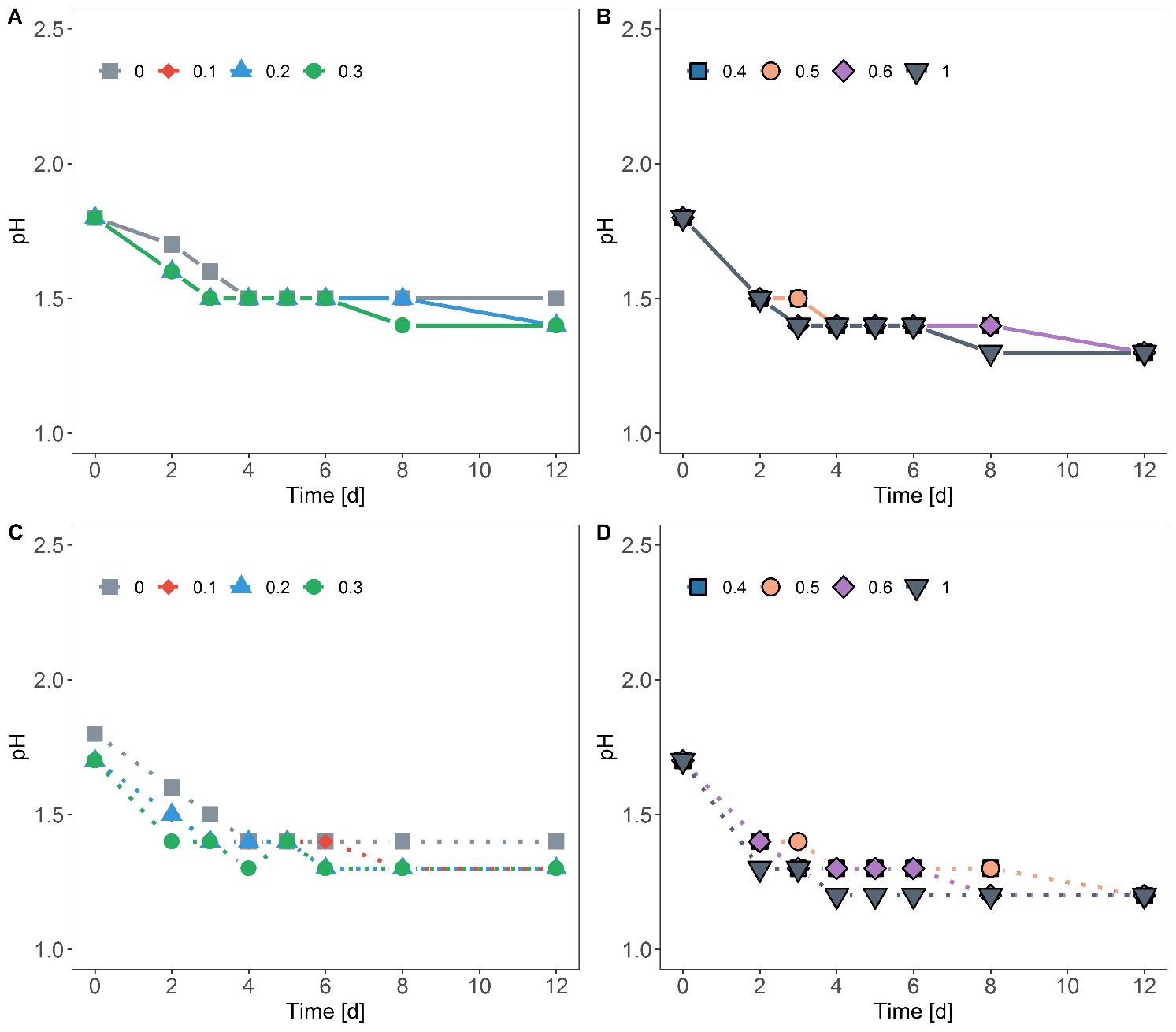


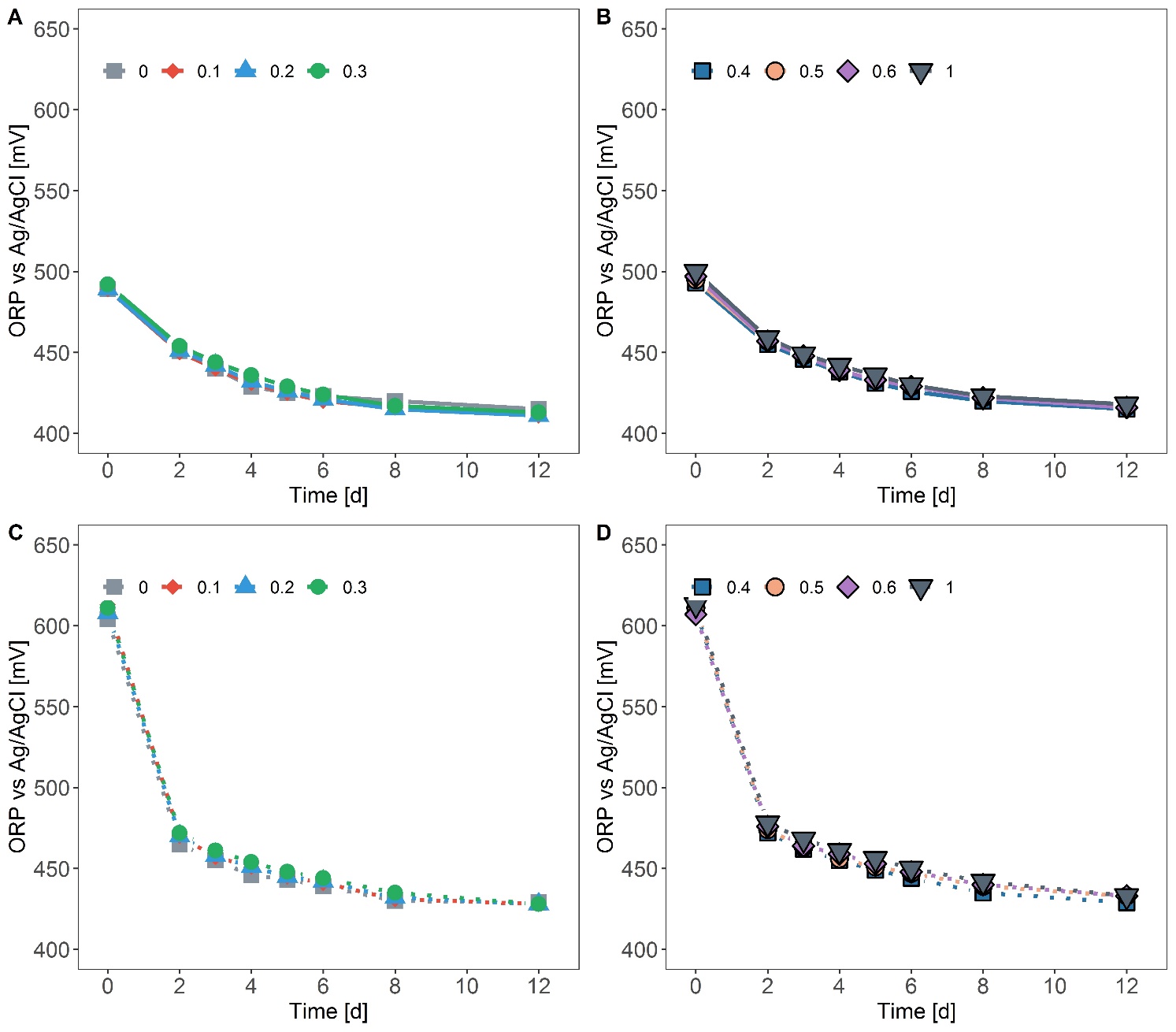


**Supplementary Figure S2.** Changes in pH and ORP (mV *vs*. Ag/AgCl) as a function of time during chemical pyrite leaching with ~ 20 mM Fe^3+^ and 30 mM Fe^2+^ (*solid line, A, B*) or ~ 40 mM Fe^3+^ (*dash line, C, D*) in the presence of NaCl concentrations. Concentrations of NaCl were 0.1, 0.2, 0.3, 0.4, 0.5, 0.6, and 1M. Values are the average of the duplicate ± deviations from the means.
